# Sub-Auroral and Mid-Latitude GNSS ROTI Performance during Solar Cycle 24 Geomagnetic Disturbed Periods: Towards Storm’s Early Sensing

**DOI:** 10.3390/s21134325

**Published:** 2021-06-24

**Authors:** Kacper Kotulak, Andrzej Krankowski, Adam Froń, Paweł Flisek, Ningbo Wang, Zishen Li, Leszek Błaszkiewicz

**Affiliations:** 1 Space Radio-Diagnostics Research Centre, University of Warmia and Mazury in Olsztyn, Oczapowski St. 2, 10-719 Olsztyn, Poland; kand@uwm.edu.pl (A.K.); adam.fron@uwm.edu.pl (A.F.); pawel.flisek@student.uwm.edu.pl (P.F.); leszekb@matman.uwm.edu.pl (L.B.); 2 Aerospace Information Research Institute (AIR), Chinese Academy of Sciences (CAS), No 9 Dengzhuang South Road, Beijing 100094, China; wangningbo@aoe.ac.cn (N.W.); lizishen@aircas.ac.cn (Z.L.)

**Keywords:** ionosphere, geomagnetic storms, GNSS, TEC, ROTI, real time

## Abstract

Geomagnetic storms—triggered by the interaction between Earth’s magnetosphere and interplanetary magnetic field, driven by solar activity—are important for many Earth-bound aspects of life. Serious events may impact the electroenergetic infrastructure, but even weaker storms generate noticeable irregularities in the density of ionospheric plasma. Ionosphere electron density gradients interact with electromagnetic radiation in the radiofrequency domain, affecting sub- and trans-ionospheric transmissions. The main objective of the manuscript is to find key features of the storm-induced plasma density behaviour irregularities in regard to the event’s magnitude and general geomagnetic conditions. We also aim to set the foundations for the mid-latitude ionospheric plasma density now-casting irregularities. In the manuscript, we calculate the GPS+GLONASS-derived rate of TEC (total electron content) index (ROTI) for the meridional sector of 10–20${}^{\circ }$ E, covering the latitudes between 40 and 70${}^{\circ }$ N. Such an approach reveals equatorward spread of the auroral TEC irregularities reaching down to mid-latitudes. We have assessed the ROTI performance for 57 moderate-to-severe storms that occurred during solar cycle 24 and analyzed their behaviors in regard to the geomagnetic conditions (described by Kp, Dst, AE, Sym-H and PC indices).

## 1. Introduction

Geomagnetic storms are not events that often pull people’s attention—they are usually noticed in the context of aurora visibility at middle or even low latitudes, while Earth’s magnetic field is an important aspect from an Earth-bound life point of view. The magnetosphere and the tightly connected ionosphere of Earth are integral parts of space weather—a complex set of solar–terrestrial interactions. The magnetosphere acts a kind of protective barrier against solar radiation and solar matter and since it interacts with energetic particles and electromagnetic radiation, it is naturally disrupted in the process.

Disruptions to Earth’s magnetic field may cause a whole range of negative effects on many aspects of everyday life [1,2]. In history, there have been reported events of magnitudes that caused severe outages in the power grid (including blackouts) and serious damages of the telegraph infrastructure [1,3]. Nowadays, humanity relies on electricity far more, and thus similar events could bring serious economic consequences for power and telecommunication systems and even extend to transport infrastructure (such as pipelines).

Many anticipated a similar extreme storm to happen in 2012, based on the observation of a chain of strong coronal mass ejections (CMEs) [4]. Eventually, the CMEs narrowly missed the Earth. Although the 2012 extreme storm in the end did not occur, such situations emphasize how important proper geomagnetic storm forecasting and now-casting are.

Infrastructure damages caused by an extreme geomagnetic storm are not only effects of the magnetosphere–ionosphere disruptions. Electromagnetic fields, (such as those that originated in the electrically charged ionosphere), may also seriously affect propagation of the radio waves [5,6]. As mentioned above, the upper, ionized part of the atmosphere—the ionosphere—is tightly connected with the geomagnetic field [7]. The presence of ionized particles inevitably connects with coexistence of the electromagnetic fields. Ionized matter and electromagnetic fields are highly disruptive media for the propagation of radio waves, causing a whole range of effects, from the scintillation of the wave intensity to the Faraday rotation (rotation of the wave polarization plane) and radio wave ray path refraction [8,9]. The ionosphere also reflects radio waves of certain frequencies (around the level of 10 MHz). The transparency of the radio waves is dependent on the state of the plasma—disturbed conditions may degrade the transparency for the low-frequency radio waves, which, in turn, is an important aspect for, e.g., low-frequency radio astronomy.

Geomagnetic field disturbances primarily impact equatorial and polar ionospheres, so the whole range of mid-latitude users should not experience ionospheric impacts, but strong events cause polar oval spread equatorward down to middle latitudes. Kotulak et al. [10] show that during high geomagnetic activity events, the auroral plasma fluctuation region extends equatorwards down to even 55${}^{\circ }$ N.

Strong geomagnetic events may seriously affect everyday aspects of life. Nowadays, Global Navigation Satellite System (GNSS) positioning is so widespread that we do not even register the presence of said systems. A number of authors have described the impact of ionospheric plasma gradients on GNSS performance, indicating that strong irregularities within the ionosphere could seriously degrade the navigation quality, accuracy and even prevent satellite positioning [11,12]. In this work, we assess the ionospheric plasma density irregularities described with Rate Of Total electron content Index (ROTI) in regard to increased geomagnetic activity.

Number of authors indicate ROTI usefulness in ionospheric irregularities studies [13,14,15]. In 2018, the International GNSS Service (IGS) introduced ROTI polar maps covering the northern hemisphere with ionospheric TEC fluctuations information [16,17]. In a previous work [10], we presented the ROTI climatological behavior and sensitivity to different solar and geomagnetic conditions over a long timescale. In this manuscript, we focus on direct ROTI responses to disturbances within the Earth’s magnetic field to assess the possibility of early detection of the storm-induced enhancements within the mid-latitude plasma density fluctuations. This work is one of the steps towards improving the IGS ROTI product reliability and approaching real-time solutions, which are of great interest for the scientific community.

The European radio interferometer LOFAR (LOw Frequency ARray) telescope is one of such scientific users of the ionospheric irregularities data products. LOFAR operates on frequencies near the border of the atmospheric radio window. Low frequencies are extremely vulnerable to all electromagnetic fields, so ionospheric plasma density gradients may seriously affect the quality of observation [6]. Błaszkiewicz et al. [18] show that even weak ionospheric total electron content (TEC) fluctuations have a strong reflection in radio pulsar observations—the observed radio wave intensity noticeably drops during occurrence of plasma density gradients in a LOFAR’s field of view.

Considering the sub- and trans-ionospheric radio transmissions’ vulnerability to the ionospheric plasma density gradients, we address the study to assess the performance of the TEC fluctuation phenomenon. In the previous study [10], we discussed, in general, the ROTI daily, seasonal, annual and solar-cycle variations. In this study, we evaluate the ROTI behavior in regard to the enhanced geomagnetic activity conditions to characterize the magnetic storms’ impact on the perturbations within the ionospheric plasma density. Our goal is to assess the ROTI response to the geomagnetic storm generation.

In the work, we also present a statistical study of storms that occurred during solar cycle 24 in terms of the ionospheric plasma behavior irregularities observed at sub-auroral and mid-latitudes.

The main objective of the work is to find key features of the storm-induced sub-auroral ionospheric plasma density irregularities to distinguish the differences in phenomena between events of different magnitude to assess the ionospheric fluctuations in terms of disruptions of Earth’s magnetic field and eventually to set the foundation for a storm-time ionosphere TEC fluctuation now-casting.

## 2. Materials and Methods

Nowadays, a whole range of tools and instruments are available to examine ionospheric plasma properties—incoherent scatter radars, ionosondes, satellite missions provided with a proper payload including, e.g., the Langmuir probe used to measure the plasma parameters in situ. Nevertheless, one of the most important and widely used is Global Positioning System (GPS) and other GNSS. Despite many limitations of that technique, GNSS are prevalent due to the global coverage, permanent 24 h monitoring time and easy, open access data (via global and regional permanent reference networks) [8,19]. In recent years, a massive GNSS-based elaboration provided us with many elaborations on the ionospheric state. Dual-frequency receivers allows the estimation of the TEC value, based on the so called geometry-free combination of observations of two different frequencies, as in Equation (1) [20]. With dual-frequency GNSS receivers, the relative STEC can be directly estimated using a geometry-free combination of GNSS observables:

(1) $$STEC_{i}=\left(\frac{L1}{f_{1}}-\frac{L2}{f_{2}}\right)*\frac{f_{1}^{2}*f_{2}^{2}}{f_{1}^{2}-f_{2}^{2}}*\frac{c}{K}$$

where *L*1 and *L*2 are carrier phase observations performed on frequencies $f_{1}$ and $f_{2}$; for the GPS, $f_{1}$ = 1525.42 MHz and $f_{2}$ = 1227.60 MHz, while GLONASS satellites frequencies vary slightly around central values of $f_{1}$ = 1525.42 MHz and $f_{2}$ = 1227.60 MHz; *c* is the speed of light in the vacuum; and *K* is a constant factor equal to 40.3.

Proper elaboration of the absolute TEC value also requires an estimation of the satellite and receiver’s biases; however, for a plasma density fluctuation detection, the relative TEC value is used in the calculation process of which the biases are eliminated. In terms of the TEC fluctuations, the Rate of TEC (ROT) is a very efficient tool. ROT is calculated as a difference of the estimated TEC values in a unit of time (usually one minute) within a single, continuous satellite-tracking arc (Equation (2)) [21,22].

(2) $$ROT=\frac{TEC_{i+1}-TEC_{i}}{t_{i+1}-t_{i}}$$

ROT can be gathered within a single, quantified measure—ROT index (ROTI). ROTI is a standard deviation of the ROT values over a chosen period and longitude–latitude cell (Equation (3)). The ROT value directly represents the exact fluctuations of the GNSS signals, whereas ROTI identifies regions with enhanced TEC value fluctuations. ROT and ROTI are expressed in TEC units (TECU) using a unit of time (minute).

(3) $$ROTI=\sqrt{\langle ROT^{2}\rangle -{\langle ROT\rangle }^{2}}$$

In our study, as we focused on latitudes of up to 70${}^{\circ }$ N, we incorporated GPS + GLONASS observations, as GLONASS would increase the elaboration performance at higher latitudes, due to its orbit’s higher inclination angle [23].

**Figure 1:**
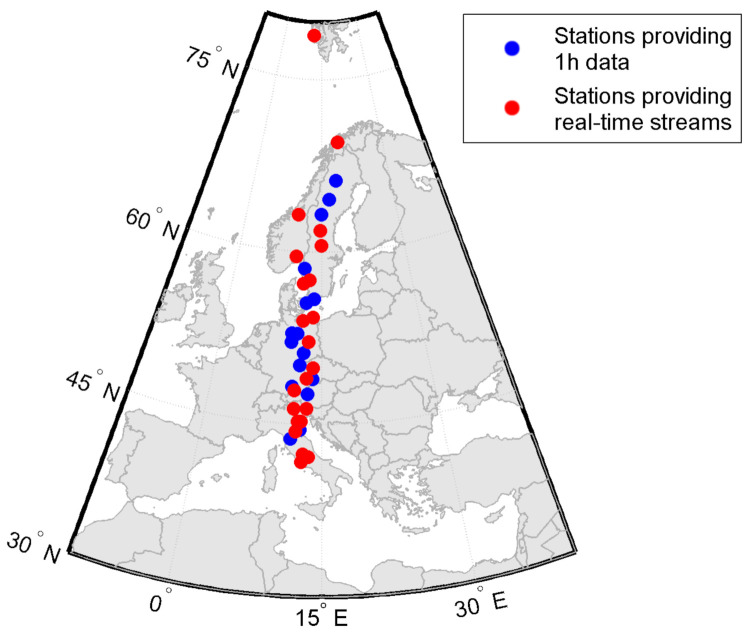
Map of the EPN stations located between 10 and 20${}^{\circ }$ E selected for meridional ROTI time series. Blue dots mark stations providing 1 h data; red ones—stations with real-time data stream access.

In a previous study [10], we have discussed the climatological behavior of ROTI in different sectors. The study revealed similar patterns that are dependent on the latitude, not longitudinal sector; therefore, we have focused on a more detailed analysis of the single sector. We have selected 40 permanent GNSS stations from the EUREF Permanent Network (EPN) distributed along single meridian (15${}^{\circ }$ E). EPN stations distribution provides a good observation coverage in the mid-latitude ionosphere. Figure 1 presents distribution of the selected EPN stations (stations with a real-time data streams are highlighted with red color). Some studies described ionospheric responses to geomagnetic storms in an approach similar to that presented here; however, these focused on the absolute TEC values [24,25].

In our previous study [10], latitudinal ROTI series were elaborated in cells fixed with 17–18 selected stations and provided a non-uniform, low resolution of three–five degrees, which allowed only a rough, general assessment. In the current work, multi-constellation observations collected at selected stations provide an Ionosphere Pierce Point (IPP, where the GNSS signal path between satellite and permanent station pierces the single thin layer Ionosphere model commonly used in IGS products) distribution that is sufficient to elaborate on ROTI in the regular grid with the latitudinal resolution of one degree.

Since our goal is to assess the ROTI behavior in the scope of geomagnetic activity, we collected the database including a whole range of events of different magnitudes, occurring during different phases of the solar cycle.

Geomagnetic storm time was distinguished with measurements of the magnetic field intensity gradients and is described with many indices. In our work, for storm identification we selected two of the most widely used ones: Kp and Dst.

The Planetary K (Kp) index was described by Bartels in 1939 [26]. It is an average value of K indices elaborated based on the observed magnetic field intensity anomalies at several observatories in different sectors of the world. The Planetary K index is calculated as a 3 h measure of the collected data and is one of the the basic detectors of disruptions within the magnetosphere. Kp is also included in the Space Weather Prediction Center of National Oceanic and Atmospheric Administration (SWPC/NOAA) web pages as an aurora visibility estimator.

Dst is elaborated using sensors distributed in low-latitude regions [27]. Dst is a detector of the IMF-triggered disruptions in Earth’s magnetic field by monitoring of the axis-symmetric signatures of the currents within the magnetosphere.

Although the Dst index is a measure of equatorial activity, it is also used in global and auroral studies of geomagnetic storms. The ring current mechanism causes the auroral magnetosphere and ionosphere to mirror strong equatorial magnetic field disruptions.

During the current solar cycle, 25 non-significant events occurred—the Kp index did not reach a value of 5, and Dst dropped below −50 nT only 5 times. Hence, we extended our study to the whole of solar cycle 24. Solar cycle 24 was the weakest since at least cycle 12 (based on the data provided by SWPC/NOAA). Solar cycle 23 was far more active and storms of any level were far more frequent—3–4 extreme events happened, in contrast to the complete lack of such events in solar cycle 24. Unfortunately, the amount of the GNSS data available before 2008 does not allow for a mid-latitude ROTI elaboration in a chosen sector with a satisfactory quality.

Table 1 presents the numbers of storms of different magnitudes that occurred during solar cycles 25, 24 and 23. Storms are classified using the Kp index, as proposed by SWPC/NOAA.

Loewe and Prölls [28] suggest similar classification of geomagnetic storms, based on the Dst index. Using that classification, strong geomagnetic occurrence during the last three solar cycles would look as presented in Table 2.

We have selected 57 moderate-to-severe events that fulfilled both Kp and Dst index criteria. Based on the Dst/Kp cumulative approach, we have distinguished 4 severe events, 15 strong ones and 38 moderate ones.

Since the Dst index is calculated over 1 h and Kp over 3 h intervals, we have incorporated them only in a general storm-time identification. For a better insight into variability and determination of the storm maximum/central point, we have used 5 min indices: AE and Sym-H.

Described by Davis and Sugiura [29], the Auroral Electrojet (AE) index is a relative measure of the distortions of the horizontal magnetic field (H). AE describes the geomagnetic disturbances generated by enhanced currents in the ionosphere below and within the auroral oval.

Sym-H is a measure of the geomagnetic irregularities within the low latitude region in terms of longitudinally symmetric magnetic field H component perpendicular to the dipole axis [30]. Sym-H describes low-latitude magnetosphere currents, and its concept is almost the same as the Dst index. Sym-H is usually treated as the Dst index for shorter intervals (down to even 1 min) [31].

For a comparison, we also selected the Polar Cap (PC) index. PC actually consists of two indices: PCN for the North Pole and PCS for the South Pole. Both indices are derived from observations obtained with two single stations (Thule for PCN and Vostok for PCS). By definition, the PC index is a measure of electric current across the polar cap [32]. The index monitors the polar cap geomagnetic activity triggered by the IMF and solar wind, driven by the geo-effective interplanetary electric field. PC is supposed to stay in close correlation with AE and Dst indices [33]. Troshichev and Janzhura [33] claim that PC responds better to the geomagnetic disturbances than raw electric field measurements since PC focuses on the part of the IMF actually impacting the magnetosphere. Therefore, PC has a potential in geomagnetic storm forecasting.

When talking about geomagnetic storms, the phenomenon of sub-storms should also be mentioned. Sub-storms are usually weaker, shorter and driven by other physical mechanisms. In general, auroral sub-storms are caused by the currents within the auroral ionosphere, whereas storms are triggered directly by the interplanetary magnetic field impact on the magnetosphere and formation of the ring current [34]. Thus, incorporation the Em-derived and IMF-triggered Sym-H and PC index allows determining whether the event is generated by the solar winds’ impact on the magnetosphere.

## 3. Results

The collected ROTI series are presented in keogram-like latitude-versus-time plots. ROTI intensity in the subsequent latitude-epoch bin is represented with a color cell which ranges from blue (ROTI = 0 TECU/min) to yellow (ROTI > 0.5 TECU/min); missing values are marked with a deep blue color. Such an approach represents the temporal evolution of the ROTI pattern simultaneously with the latitudinal variability.

The presented ROTI series were calculated within the one degree of the latitudinal step with a slightly extended longitudinal window (five degrees) in order to gather more data. We applied 5 min intervals for ROTI calculation. ROTI elaboration with 30 s data and 5 min averaging time provides a good sensitivity to the perturbations within the ionospheric plasma density [35].

### 3.1. Selected GNSS ROTI Compared with Geomagnetic Activity

Since we have elaborated on ROTI for 57 events, in the manuscript we have included only a few of the most representative cases (Table 3); however, we will still discuss the behavior of all the events. We include all four severe storms and two examples for strong and moderate events.

**Figure 2:**
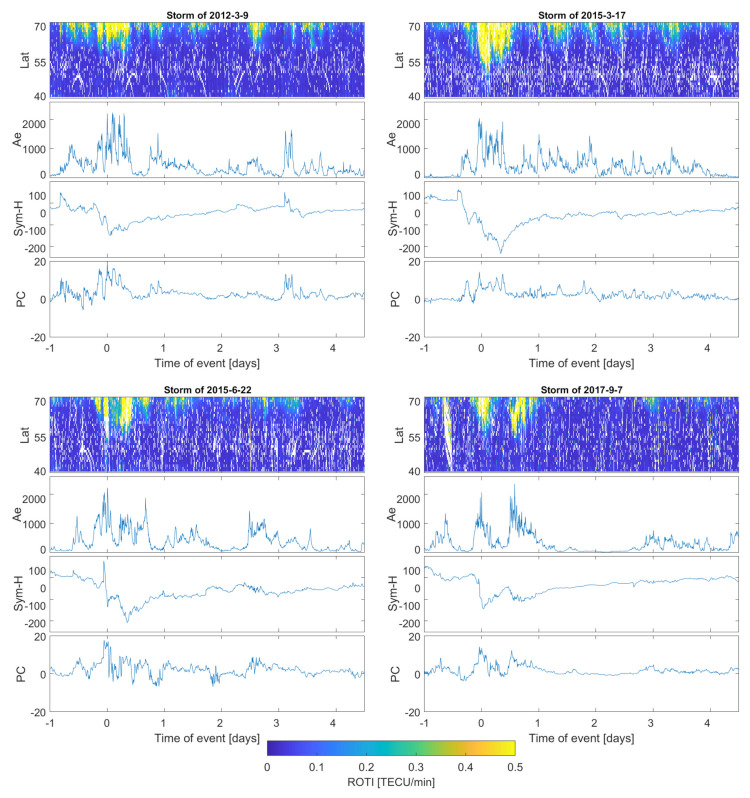
ROTI two−dimensional time series for severe geomagnetic storms (9 March 2012, 17 March 2015, 22 June 2015 and 7 September 2017) compared with Ae, Sym-H and PC indices series.

**Figure 3:**
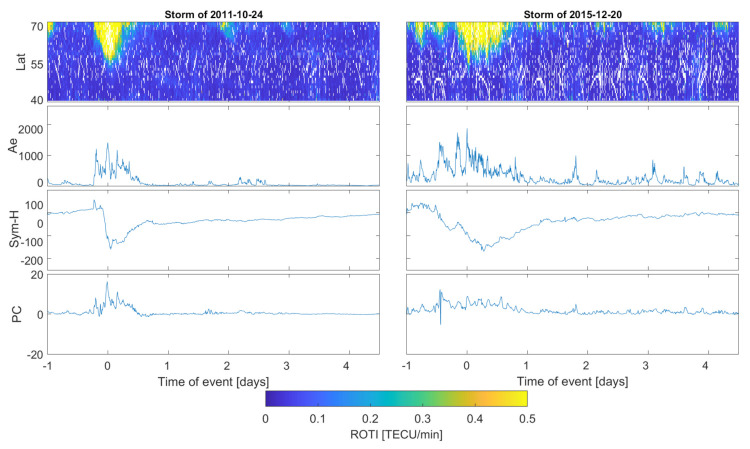
ROTI two−dimensional time series for strong geomagnetic storms (24 October 2011, and 12 December 2015) compared with Ae, Sym-H and PC indices series.

**Figure 4:**
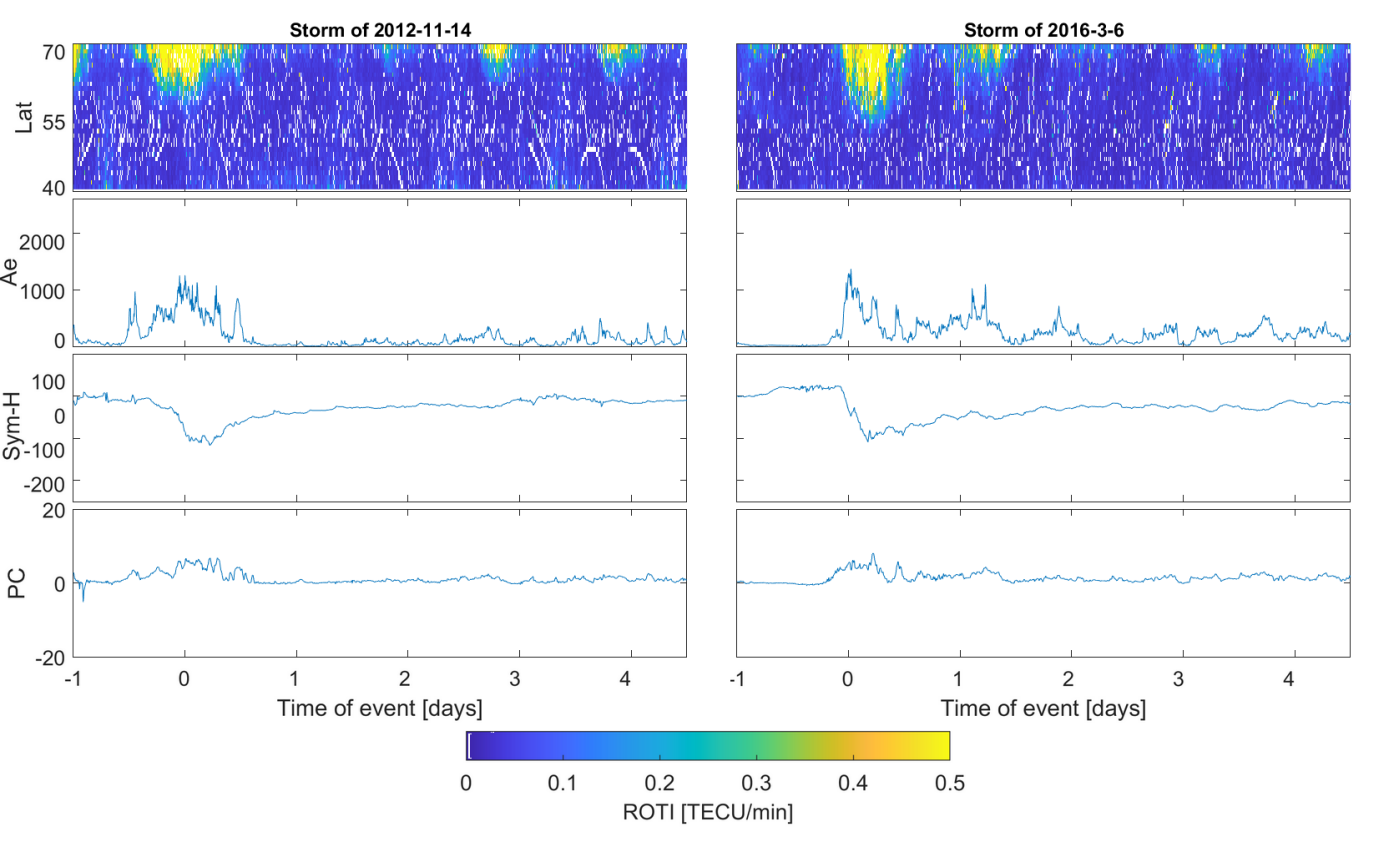
ROTI two−dimensional time series for moderate geomagnetic storms (11 November 2012 and 3 March 2016) compared with Ae, Sym-H and PC indices series.

Figure 2, Figure 3 and Figure 4 present stacked plots of the ROTI, AE, Sym-H and PC index series for severe, strong and moderate storms, respectively. All data series are centered on the storm maximum point estimated with regard to the maximum value of AE and minimum of the Sym-H value. Krankowski et al. [36], in a study of auroral irregularities, divided ROTI into two ranges: between 0.3 and 0.5 and above 1.5 TECU/min. Thus, in our study we applied the ROTI of 0.4 TECU/min threshold. Such an approach would also result in a better readability of the color-scaled two-dimensional ROTI series. Sub-auroral ROTI values during serious events usually exceeds the level of 1.00 TECU/min, reaching the maximum level of a few TECU/min. For a proper assessment of whole event, the data series also includes the 24 h before the storm’s peak and the 4-day period after the main phase ended. We have also computed ROTI series for a pre-storm quiet time reference with a Kp below 3 and Dst not decreasing below −30 nT. Quiet time ROTI analysis did not reveal any spread of the high irregularities below 70${}^{\circ }$ N; therefore, all high sub-auroral ROTI occurrences seem to be connected with disturbances in geomagnetic conditions.

All severe storms have complex natures with weaker sub-storms occurring before the main phase. Additionally, for the main phase for 22 June 2015, and 7 September 2017, the storms seem to be split into two sub-maxima separated by several-hour intervals. Additionally, the geomagnetic indices reveal the storms structures’ complexity—the pre-storm shock can be clearly seen in the Ae values increasing to 1000 nT right before the main phase increase (to values above 2000 nT). A similar behavior can be observed within the Sym-H index series. In all presented storms, multiple "sub-drops" in Sym-H values can be distinguished. Previous studies [24,28] describe similar complex behaviors of the geomagnetic storms and distinguished similar multiple-shock pattern with prolonged depletion in Dst and Sym-H values. During severe storms, the maximum Sym-H index dropped to the level of −200 nT (and even below for the 17 March 2015 storm). In all four presented cases, the event was accompanied by a sharp PC index variability.

During main phase high ROTI occurrence, the region expanded equatorward down to 55${}^{\circ }$ N (even lower—to 52${}^{\circ }$ N—during the 17 March 2015 storm). The main phase lasted 12 h up to whole day. During the recovery phases of storms, some sub-storms (with AE reaching level of 1000 nT) often occurred with a respective equatorward spread of auroral ROTI to the latitude of 65${}^{\circ }$ N.

ROTI-enhanced values within the storm-extended auroral oval correlate well with the geomagnetic indices with correlation coefficient above 0.5 for all the cases.

The case of the 24 October 2011 event is noticeably weaker in terms of geomagnetic indices. AE reached a value of 1500 nT and Sym-H dropped to −150 nT. The PC index revealed storm-indicating variability; however, its variation was weaker than during severe storms.

Among the 15 strong storms, two different ROTI spread patterns could be identified: (a) a sharp spread towards 55${}^{\circ }$ N with a duration of up to several hours and (b) an extended spread lasting 12 h and more but reaching latitudes of 60–65${}^{\circ }$ N. During all registered events, the AE values usually oscillated around the level of 1000 nT, with occasional single spikes to the 2000 nT levels.

The cases of the 20 December 2015 (strong) and 9 March 2012 (severe) storms reveal very similar patterns and intensities in a matter of high ROTI sub-auroral spread. Both storms are characterized by similar Dst and Sym-H behaviors reaching similar minima. Such strong ROTI are due to weaker magnetic conditions occasionally, but this is not a rule.

Moderate storms can be characterized similarly as strong events, as within 37 analyzed cases, we found similar divisions into two kinds: stronger but shorter spreads, reaching lower latitudes, and longer-lasting ones, with an equatorward spread to around 60–65${}^{\circ }$ N. The enhanced ROTI occurrence duration is similar to strong storms which occurred for several hours for single ROTI spikes and 12 h up to whole day for the prolonged structures.

Statistically, the ROTI sub-auroral spread reaches higher latitudes; however, sometimes can extend even down to 55${}^{\circ }$ N (as during the 5 March 2015 storm (Figure 4), with AE achieving the maximum level of slightly above 1000 nT and Sym-H dropping only to −100 nT. Therefore, it allows us to conclude that sub-auroral TEC fluctuations spread range is limited to the latitude of 55${}^{\circ }$ N and presence of even weaker magnetic field disturbances is sufficient for plasma density irregularities to occur at lower latitudes. Additionally, similarities in patterns revealed during storms of different magnitudes confirms such a conclusion. However, a lower ROTI spread is more frequent and ROTI reaches noticeably higher values during stronger events; therefore, a stronger ROTI response to a weaker storm should be treated as an extraordinary behavior.

None of analyzed 57 moderate-to-severe events revealed any usual sub-auroral irregularities, besides occasional substorm occurrences. ROTI spread is typical only for periods of the directly disturbed conditions (exact shocks within the magnetosphere).

### 3.2. GNSS ROTI Validation with Swarm In Situ Measurements

In order to validate the ROTI performance, we have included Swarm satellite Langmuir probe in situ measurements for reference with an independent instrument observations. In order to do this, we incorporated the method described by Zakharenkova et al. [37]. Swarm satellite mission consists of three spacecrafts: Swarms A and C are set on an orbit of 460 km altitude whereas Swarm B is on a 500 km orbit. Each spacecraft payload is provided with a GNSS receiver and Langmuir probe. Satellite orbits have 87–88° inclination angles, so the observations cover almost the whole latitudinal range. In the first step, we have selected Swarm satellites’ passages over the selected region (10–20${}^{\circ }$ E and 40–70${}^{\circ }$ N) with time collocated with the duration of the geomagnetic storm. For the selected Swarm passes, we collected the measured electron density (Ne) and normalized it to 10${}^{6}$ electron/cm${}^{3}$. Similarly to ROT/ROTI, we calculated the Rate of Density (ROD) and Rate of Density Index (RODI)—ROD as a temporal variation of the normalized Ne and RODI as a standard deviation of a continuous set of ROD values.

**Figure 5:**
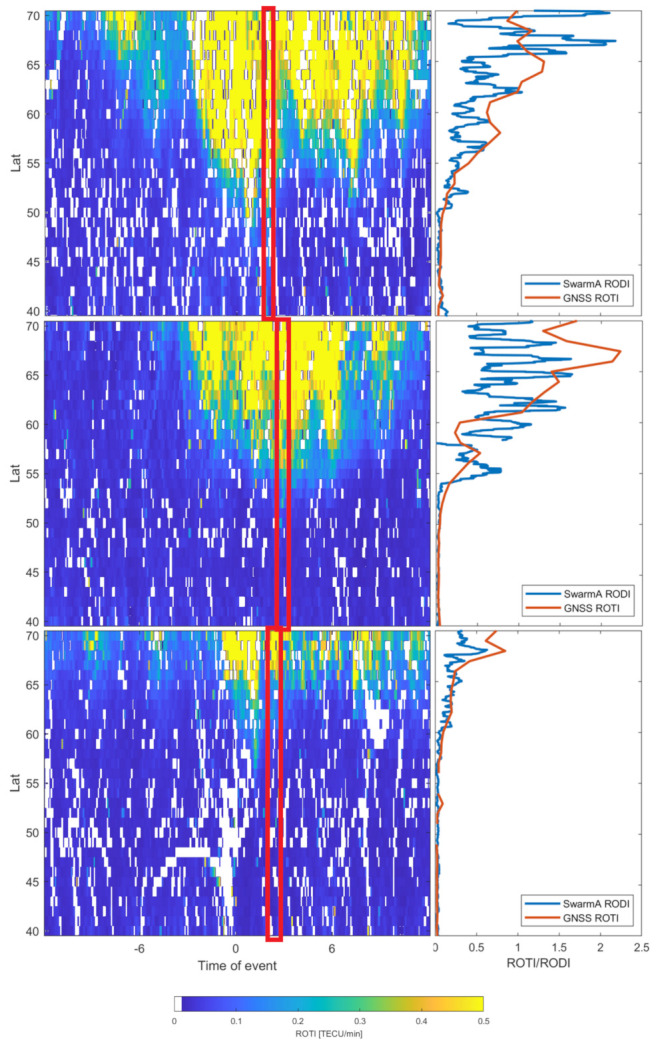
Selected examples of GNSS ROTI validation with RODI data derived from Swarm in situ plasma density measurements. Cases of 17 March 2015, 6 March 2016 and 11 September 2015, respectively.

Selected results of GNSS ROTI/Swarm RODI validation are presented in Figure 5. A window in the storms two-dimensional series marks the time it took Swarm A satellite to pass above the longitudinal 10–20${}^{\circ }$ E band. The linear plot presents the latitudinal profile of GNSS ROTI in that particular moment compared with Swarm-derived RODI. The GNSS ROTI profile has been calculated as an average from a 15 min period (three epochs marked with red box in Figure 5), since the Swarm spacecraft’s passage over the studied area lasts around 10 min.

In order to compare GNSS ROTI and Swarm RODI measurements, we also calculated the correlation coefficients, mean square errors (MSE) and mean average errors (MAE) for each case Table 4. Despite some visible differences between ROTI and RODI series, the correlation between those can be considered as high—for all the cases correlation coefficient exceeds 0.7.

**Figure 6:**
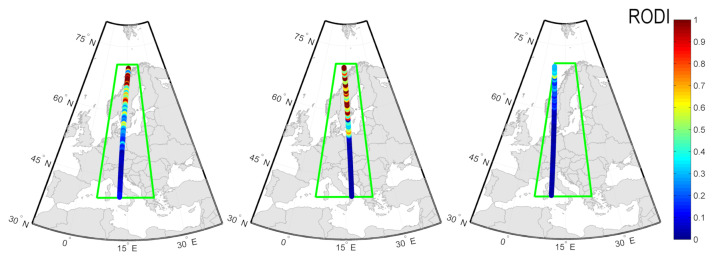
Swarm RODI measurements and positions for the selected cases, respectively, within the study area (green box).

Figure 6 presents the Swarm RODI measurements for subsequent cases within the region selected for the study.

One can spot some differences between the ROTI and RODI structures during stronger events; however, it should be remembered that GNSS and Swarm observations have different spatial resolutions and observation intervals, which may result in different sensitivities depending on the structures’ scale and velocity [35]. Additionally, space-borne observations obtained with Swarm are tied to the orbit altitude and therefore may not reveal all structures [38].

Although Swarm spacecrafts’ coverage of the selected region during the geomagnetic storms’ main phases was relatively poor (for each storm, there was up to one corresponding Swarm passage), the validation data still confirm the ROTI sensitivity to plasma density irregularities. The general latitudinal structure reveals similar behaviors both in GNSS ROTI and Swarm RODI, regardless the magnitude of the event.

## 4. Discussion

During a magnetic storm, a whole range of effects may occur within the sub-auroral ionosphere. Prölls et al. [39] describe four different types of both positive and negative effects of plasma density. The ROTI discussed in this work, however, by its calculation method, is sensitive both to rapid jumps and drops in the total electron content. ROTI is calculated as a standard deviation of the temporal TEC fluctuations and thus always takes values greater than zero. High values may correspond to strong positive and negative gradients of ROT.

Our work confirms that the equatorward spread of the auroral zone of the increased TEC fluctuations described with ROTI is a typical feature of geomagnetic storms of different magnitudes [10,40].

**Figure 7:**
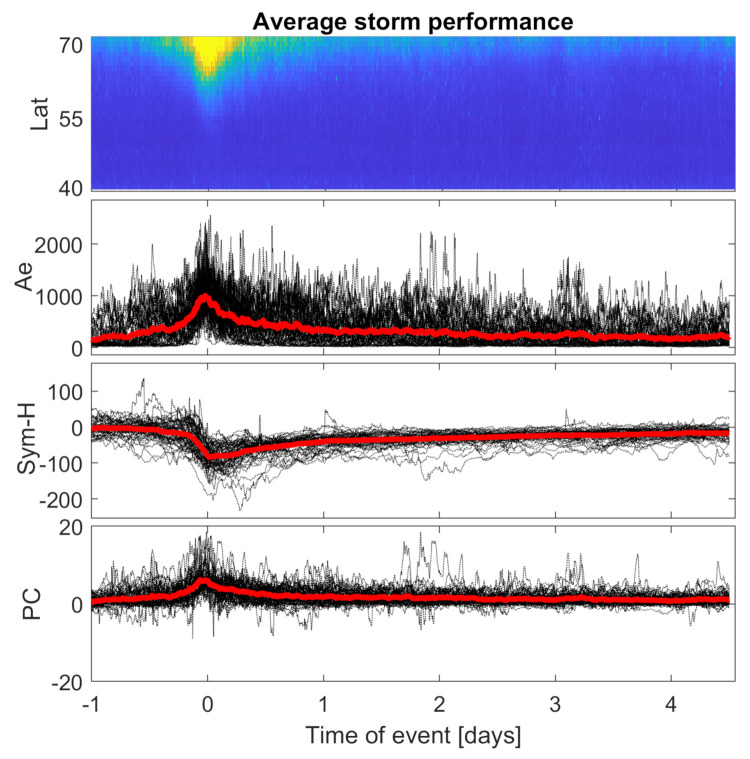
Average storm−time ROTI performance in comparison with averaged AE, Sym-H and PC indices.

In order to check the average behavior of ROTI described fluctuations, we calculated the mean value of the storm maximum-centered ROTI series (Figure 7).

However, the average ROTI storm behavior should not be treated as a model of any kind, and some of its features can give some information on the general patterns. Most of the analyzed strong/moderate storms follow a similar pattern in terms of ROTI behavior, as well as in terms of indices’ performances.

Any extraordinary characteristics of single events are smoothed out. Every single event of course has a unique structure; however, the smoothed average reveals a few features of the "typical" storm behavior and gives an insight into how the minimal expectation of ionospheric irregularities should look like to be treated as a storm-time indicator. The obtained, averaged storm pattern is similar to the mean geomagnetic storm behavior described by Loewe and Proölls [28].

Increased ROTI auroral oval expanding towards mid-latitudes is sharper than its recovery to the quiet time behavior. The spread sawtooth-like structure resembles the Sym-H index drop during the main phase of the event. Substorms’ equatorward ROTI spreads are more frequent in the storm preparation phase and during the first 24 h after a storm’s peak. Although the recovery phase lasts for few days after the main phase, ROTI does not reveal any sub-auroral increase.

Statistical study of storms reveals that, during storms, the ROTI spread range is typically dependent on the event magnitude and reaches 65${}^{\circ }$ N, 60${}^{\circ }$ N and 55${}^{\circ }$ N for moderate, strong and severe storms, respectively; the maximum latitudinal range of 52${}^{\circ }$ N can be reached during storms of any magnitude (Table 5). Table 5 also shows that the situation looks different in the context of ROTI values. ROTI consistently reaches higher values for stronger magnetic disturbances.

Based on the severe storms, we have calculated a cross-correlation between ROTI and AE, Sym-H and PC indices in order to assess the time interdependency between ROTI and indices’ performances. General correlation between ROTI and geomagnetic parameters have been described before [10]; therefore, we have focused on the exact storm occurrence time.

**Figure 8:**
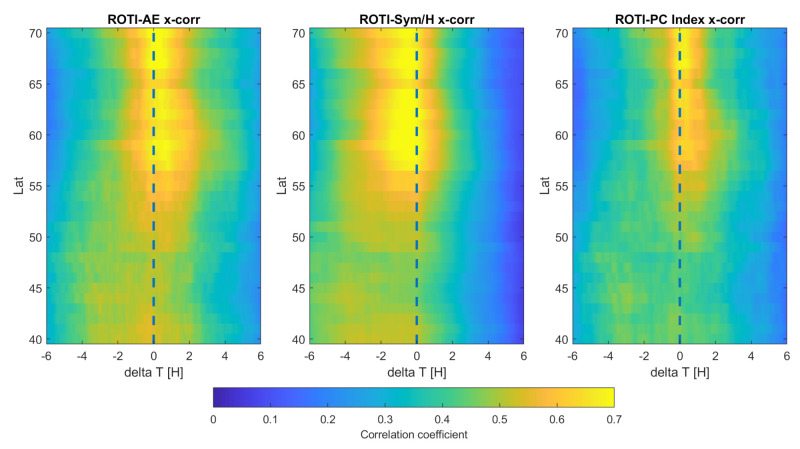
Cross−correlation pattern between ROTI and AE, Sym-H, PC indices.

The cross-correlation analysis between ROTI and geomagnetic activity indices (Figure 8 revealed a delay pattern that corresponds well with the ring current formation—the ROTI sub-auroral spread is preceded by a Sym-H drop (for around 0.5 h). The time lag is caused by the time required by the ionosphere to respond to solar wind-induced disruptions of the magnetic field’s horizontal component. ROTI-AE cross-correlation reveals the opposite—the AE pattern is delayed for about 10–30 min with regard to the ROTI. This corresponds well with the definition of the AE index, as a measure of magnetic field disruptions caused by the ionospheric plasma irregularities.

ROTI-PC cross-correlation reveals the maximum correlation of both series for around-zero lag value. This confirms that ionospheric TEC fluctuations sub-auroral spread is directly connected with the increased geomagnetic activity within the auroral oval.

Additionally, one can clearly see that all indices correlate well with ROTI at latitudes down to 55${}^{\circ }$ N—similar to the previous study. ROTI also reveals a slightly stronger correlation with the AE index (with correlation coefficient reaching 0.8 in comparison to a maximum of 0.7 for correlations with Sym-H and PC indices), which appears to be legitimate, as AE directly addresses the ionospheric activity disturbances within the auroral zone. Correlation drops at lower latitudes and so is outside the storm-extended auroral oval. As we presented in the previous work, also addressing the IGS northern-hemisphere ROTI product, the enhanced ROTI zone extent corresponds to models of the auroral oval shape [10].

In our work, we also aimed to set a foundation for a future storm-induced ROTI now-casting. All presented cases were elaborated with use of the EPN daily RINEX data, as the study was based on the historical SC24 events. In terms of now-casting, the problem of data availability and latency is extremely crucial. Daily data are available after the end of each day, so only storms that started shortly before midnight could be properly now-casted. ROTI elaboration based on the daily data provided by the permanent reference networks (such as IGS or EPN) will in many cases result in serious delay—only storms that started close to midnight would be detected fast enough to allow us to consider now-casting. Since the main phase of the storm lasts up to 12 h (especially during moderate and strong events), storms starting before noon would be registered after the main phases ended. Including latency in data availability, ROTI would most often miss the beginning of the storm by more than 10 h.

**Figure 9:**
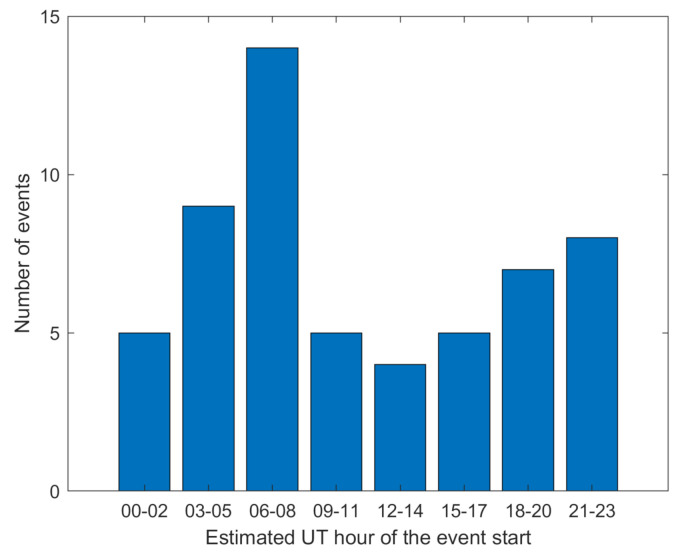
Histogram of the estimated UT hour of the geomagnetic storm beginning over three-h intervals.

Figure 9 presents the histogram of the analyzed events start time. The conclusion is simple—storms may begin at any time; therefore, in most cases daily GNSS data would be available too late.

Nowadays, permanent reference networks (including EPN) hopefully provide data with a shorter collecting time and latency. Most of the EPN stations provide hourly and 15 min intervals, shortening the now-casting latency from >10 h to around 1. All stations selected in this study provide hourly data with a 20 min data availability delay.

**Figure 10:**
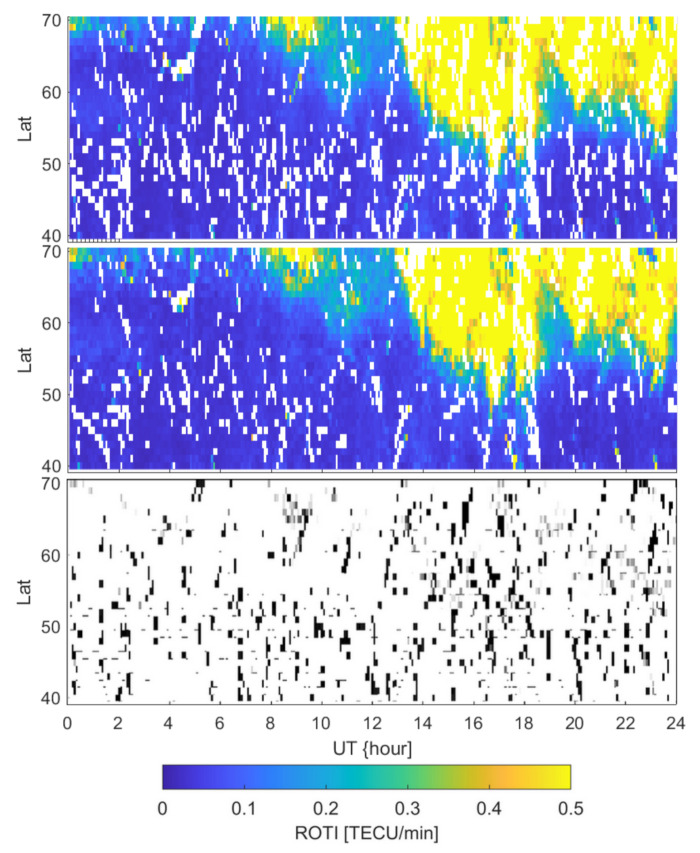
Comparison between ROTI obtained with only 23 stations broadcasting real-time streams (top panel) and all 40 selected stations (middle panel) for a day of the storm (case of 17 March 2015). The bottom panel presents residuals with black pixels indicating extra blank spots in the solution based only on the stations providing real-time data streams.

What is more, almost a third of the EPN permanent GNSS stations broadcast real-time data streams. Among the 40 selected in this study, 23 are characterized by such an availability. Figure 1 also presents the distribution of stations with a real-time streams’ availability. In our study, we used the data from all available stations rather than only those providing real-time data streams, as the number of the latter is noticeably lower. Fewer stations results in a noticeable degradation of obtained ROTI series (Figure 10).

However, the general occurrence of the enhanced ROTI can be spotted in both cases; the blank spots occurs significantly more frequently for observations collected with a limited number of stations. Blank spots in both cases occur in crucial parts of the series; however, in a real-time-only observations case, the lacking data noticeably degrade the structure detectability.

## 5. Conclusions

Within our study, we have examined 57 moderate-to-severe geomagnetic storms in the scope of ionospheric TEC irregularities within the mid-latitudes to find general features of storm-enhanced ROTI behavior.

ROTI sub-auroral spread towards mid-latitudes occurred in every analyzed study. Strong TEC fluctuations expand down to 55${}^{\circ }$ N latitude. The maximum spread range can be also reached during events of lower magnitudes. However, weaker storms are accompanied by weaker and shorter ROTI equatorward spreads.

ROTI mid-latitude occurrences correlate well with parameters of geomagnetic activity. Cross-correlation between ROTI series and AE, Sym-H and PC reveals strong correlation with auroral processes. Lags in maximum correlation between ROTI and the indices indicates that TEC fluctuations’ spread may be driven by the formation of the ring current.

Although the data collected from 40 selected stations seem sufficient to determine the general storm-time ROTI behavior, a closer look reveals that the obtained series suffers from occasional occurrence of blank spots. In this study, we based ROTI calculation on GPS+GLONASS data. Inclusion of other systems (GALILEO, BEIDOU, QZSS) would provide a better observation coverage, especially in a matter of real-time elaboration, where the amount of data is significantly lower due to the real-time streams’ availability.

During stronger events, ROTI reaches higher values (1.9 for severe storms, 1.3 for strong and 0.9 for moderate) and typically spreads further equatorwards, but occasionally in terms of >0.4 TECU/min, similar ROTI spread patterns can be observed during more or less disturbed periods. During stronger events, ionospheric disturbances basically last longer.

ROTI behavior stays in clear correspondence not only with the geomagnetic activity index, but also with independent in situ plasma fluctuation measurements.

In general, sub-auroral ROTI spread detection is a very promising sensor for the now-casting of geomagnetic storms. We also show that storm-induced ionospheric gradients should be also carefully taken into account with radio-based studies settled within the mid-latitudes, as fluctuations’ spread may reach latitudes down to even 52${}^{\circ }$ N.

Our study shows that even regional ROTI elaboration within a narrow sector and with a limited number of GNSS receivers can reveal some characteristics of the storm-induced ionospheric plasma density irregularities and equatorward spread of the auroral oval. As we presented, currently the number of GNSS permanent stations with a real-time stream access is sufficient for some elaborations; however, the studies benefit a lot from incorporating, e.g., hourly data available with about half an hour latency.

Dense regional GNSS permanent networks provide a good perspective for a complement to ROTI products, especially from many scientific users’ points of view. Additionally, the IGS Ionospheric Working Group’s objectives would benefit from further development of reliable, rapid, real-time-approaching solutions.

## Figures and Tables

**Table 1:** Geomagnetic storms classification based on the Kp index.

Storm Strength (Kp)	SC25	SC24	SC23
Extreme (Kp = 9)	0	0	3
Severe (Kp = 8)	0	2	31
Strong (Kp = 7)	0	26	60
Moderate (Kp = 6)	0	133	236
Minor (Kp = 5)	0	370	543

**Table 2:** Geomagnetic storms classification based on the Dst index.

Storm Strength (Kp)	SC25	SC24	SC23
Great (Dst < −350 nT)	0	0	4
Severe (Dst < −200 nT)	0	3	20
Strong (Dst < −100 nT)	0	15	98
Moderate (Dst < −50 nT)	5	58	460
Weak (Dst < −30 nT)	77	195	967

**Table 3:** Geomagnetic storm classification based on the Dst index.

Date	Storm Magnitude	Kp${}_{max}$	Dst${}_{min}$ [nT]
2011-10-24	Strong	7	−79
2012-03-09	Severe	8	−145
2012-11-14	Moderate	6+	−104
2015-03-17	Severe	8−	−223
2015-06-22	Severe	8+	−204
2015-12-20	Strong	7−	−155
2016-03-06	Moderate	6+	−98
2017-09-07	Severe	8+	−142

**Table 4:** Correlation coefficient, MSE and MAE between GNSS ROTI and Swarm RODI for selected cases.

	Case 1	Case 2	Case 3
Date	2015-03-17	2016-03-06	2015-09-11
Correlation	0.7088	0.9091	0.7533
MSE	0.1266	0.0130	0.2376
MAE	0.2245	0.0568	0.2889

**Table 5:** Typical and maximum features of storm-induced ROTI spread.

Event Magnitude	Maximum Spread Range	Average Spread Range	Maximum ROTI Peak	Average ROTI Peak
Severe	52${}^{\circ }$ N	55${}^{\circ }$ N	5.92	1.92
Strong	52${}^{\circ }$ N	60${}^{\circ }$ N	3.92	1.39
Moderate	52${}^{\circ }$ N	65${}^{\circ }$ N	3.01	0.91

## Data Availability

The study was based on the RINEX observation files obtained from the EUREF Permanent Network (ftp://epncb.oma.be/pub/, accessed on 1 March 2021), precise orbit products provided by the International GNSS Service (ftp://gdc.cddis.eosdis.nasa.gov, accessed on 1 March 2021), Swarm mission observations obtained from (https://earth.esa.int/eogateway/missions/swarm, accessed on 15 March 2021) database, geomagnetic information obtained via SWPC/NOAA (https://www.swpc.noaa.gov, accessed on 8 March 2021) and NASA/GSFC’s Space Physics Data Facility’s OMNIWeb service (https://omniweb.gsfc.nasa.gov, accessed on 8 March 2021).

## Abbreviations

The following abbreviations are used in this manuscript:

MDPI	Multidisciplinary Digital Publishing Institute
CME	Coronal Mass Ejection
EPN	Euref Permanent Network
GLONASS	GLObalnaja NAwigacionnaja Sputnikowaja Sistiema
GNSS	Global Navigation Satellite System
GPS	Global Positioning System
IGS	International GNSS Service
IMF	Interplanetary Magnetic Field
LOFAR	LOw Frequency ARray
ROD	Rate Of Density
RODI	Rate Of Density Index
ROT	Rate Of TEC
ROTI	Rate Of TEC Index
SWPC	Space Weather Prediction Center
NOAA	National Oceanic and Atmospheric Administration
TEC	Total Electron Content

## References

[1] Boteler D.H. (1991). Predicting geomagnetic disturbances on power systems. Eos Trans..

[2] Davies K. (1990). Ionospheric Radio.

[3] Green J.L., Boardsen S., Odenvald S., Humble J., Pazamickas K.A. (2006). Eyewitness reports of the great auroral storm of 1859. Adv. Space Res..

[4] Liu Y., Luhmann J., Kajdič P. (2014). Observations of an extreme storm in interplanetary space caused by successive coronal mass ejections. Nat. Commun..

[5] Béniguel Y., Angling M., Banfi E., Bourga C., Cueto M., Fleury R., García-Rigo A., Hamel P., Hartmann R., ez-Pajares M.H. Ionospheric Effects on GNSS Performance. Proceedings of the Ionospheric Effects on GNSS Performance 2012 6th ESA Workshop on Satellite Navigation Technologies (Navitec 2012) and EuropeanWorkshop on GNSS Signals and Signal Processing.

[6] De Gasperin F., Mevius M., Rafferty D.A., Intema H.T., Fallows R.A. (2018). The effect of the ionosphere on ultra-low-frequency radio-interferometric observations. Astron. Astrophys..

[7] Newell P.T., Greenwald R.A., Ruohoniemi J.M. (2001). The role of the ionosphere in aurora and space weather. Rev. Geophys..

[8] Hernández-Pajares M., Juan J.M., Sanz J., Aragón-Àngel A., García-Rigo A., Salazar D., Escudero M. (2011). The ionosphere: Effects, GPS modeling and the benefits for space geodetic techniques. J. Geod..

[9] Prikryl P., Ghoddousi-Fard R., Weygand J., Viljanen A., Connors M., Danskin D., Jayachandran P.T., Jacobsen K., Andalsvik Y., Thomas E. (2016). GPS phase scintillation at high latitudes during the geomagnetic storm of 17–18 March 2015. J. Geophys. Res. Space Phys..

[10] Kotulak K., Zakharenkova I., Krankowski A., Cherniak I., Wang N., Fron A. (2020). Climatology Characteristics of Ionospheric Irregularities Described with GNSS ROTI. Remote Sens..

[11] Astafyeva E., Yasyukevich Y., Maksikov A., Zhivetiev I. (2014). Geomagnetic storms, super-storms, and their impacts on GPS-based navigation systems. Space Weather.

[12] Basu S., Basu S., Makela J., MacKenzie E., Doherty P., Wright J., Rich F., Keskinen M., Sheehan R., Coster A. (2008). Large magnetic storm-induced nighttime ionospheric flows at midlatitudes and their impacts on GPS-based navigation systems. J. Geophys. Res. Space Phys..

[13] Aarons J., Lin B. (1999). Development of high latitude phase fluctuations during the 10 January, 10–11April, and 15 May 1997 magnetic storms. J. Atmos. Sol. Terr. Phys..

[14] Li Q., Zhu Y., Fang K., Fang J. (2020). Statistical Study of the Seasonal Variations in TEC Depletion and the ROTI during 2013–2019 over Hong Kong. Sensors.

[15] Cherniak I., Zakharenkova I., Sokolovsky S. (2019). Multi-instrumental observation of storm-induced ionospheric plasma bubbles at equatorial and middle latitudes. J. Geophys. Res. Space Phys..

[16] Cherniak I., Krankowski A., Zakharenkova I. (2014). Observation of the ionospheric irregularities over the Northern Hemisphere: Methodology and service. Radio Sci..

[17] Cherniak I., Krankowski A., Zakharenkova I. (2018). ROTI Maps: A new IGS ionospheric product characterizing the ionospheric irregularities occurrence. Gps Solut..

[18] Błaszkiewicz L.P., Flisek P., Kotulak K., Krankowski A., Lewandowski W., Kijak J., Froń A. (2021). Finding the Ionospheric Fluctuations Reflection in the Pulsar Signals’ Characteristics Observed with LOFAR. Sensors.

[19] Jakowski N., Béniguel Y., De Franceschi G., Hernández-Pajares M., Jacobsen K.S., Stanislawska I., Tomasik L., Warnant R., Wautelet G. (2012). Monitoring, tracking and forecasting ionospheric perturbations using GNSS techniques. J. Space Weather Space Clim..

[20] Hofmann-Wellenhof B., Lichtenegger H., Collins J. (2001). Global Positioning System. Theory and Practice.

[21] Pi X., Mannucci A.J., Lindqwister U.J. (1997). Ho CM Monitoring of global ionospheric irregularities using the worldwide GPS network. Geophys. Res. Lett..

[22] Wanniger L., Gendt G., Dick G. (1995). Monitoring ionospheric disturbances using IGS Network. IGS Workshop Proceedings, Special Topics and New Direction.

[23] Cherniak I., Zakharenkova I. (2017). New advantages of the combined GPS and GLONASS observations for high-latitude ionospheric irregularities monitoring: Case study of June 2015 geomagnetic storm. Earth Planets Space.

[24] Krankowski A., Shagimuratov I.I., Baran L.W. (2007). The structure of the mid- and high-latitude ionosphere during the November 2004 storm event obtained from GPS observations. Acta Geophys..

[25] Krankowski A., Shagimuratov I.I., Baran L.W., Yakimova G. (2004). Storm-time structure and dynamics of the ionosphere obtained from GPS observations. Artif. Satell..

[26] Bartels J., Heck N.H., Johnston H.F. (1939). The three-hour-range index measuring geomagnetic activity. J. Gheophys. Res..

[27] Sugiura M. (1964). Hourly values of equatorial Dst for the IGY. Ann. Int. Geophys..

[28] Loewe C.A., Prölss G.W. (1997). Classification and mean behavior of magnetic storms. J. Geophys. Res..

[29] Davis T.N., Sugiura M. (1966). Auroral electrojet activity index AE and its universal time variation. J. Geophys. Res..

[30] Iyemori T. (1990). Storm-time magnetospheric currents inferred from mid-latitude geomagnetic field variations. J. Geomag. Geoelectr..

[31] Wanliss J.A. When is it alright to use SYM-H as a storm index?. Proceedings of the AGU Fall Meeting.

[32] Troshichev O.A., Dmitrieva N.P., Kuznetsov B.M. (1979). Polar cap magnetic activity as a signature of substorm development. Planet. Spcae Sci..

[33] Troshichev O.A., Janzhura A. (2009). Relationship between the PC and AL indices during repetitive bay-like magnetic disturbances in the auroral zone. J. Atmos. Sol. Terr. Phys..

[34] Lakhina G.S., Alex S., Mukherjee S., Vichare G. On magnetic storms and substorms. Proceedings of the ILWS Workshop.

[35] Jacobsen K.S. (2014). The impact of different sampling rates and calculation time intervals on ROTI values. J. Spacea Weather. Space Clim..

[36] Krankowski A., Shagimuratov I., Baran L., Ephishov I. (2005). Study of TEC fluctuations in Antarctic ionosphere during storm using GPS observations. Acta Geophys. Pol..

[37] Zakharenkova I., Astafyeva E., Cherniak I. (2016). GPS and in situ Swarm observations of the equatorial plasma density irregularities in the topside ionosphere. Earth Planet Space.

[38] Zakharenkova I., Cherniak I., Krankowski A. (2019). Features of storm-induced ionospheric irregularities from ground-based and spaceborne GPS observations during the 2015 St. Patrick’s Day Storm. J. Geophys. Res. Space Phys..

[39] Prölss G.W., Brace L.H., Mayr H.G., Carignan G.R., Killeen T.L., Klobuchar J.A. (1991). Ionospheric storm effects at subauroral latitudes: A case study. J. Geophys. Res..

[40] Cherniak I., Zakharenkova I. (2015). Dependence of the high-latitude plasma irregularities on the auroral activity indices: A case study of 17 March 2015 geomagnetic storm. Earth Planets Space.

